# Role of advanced glycation end products in mobility and considerations in possible dietary and nutritional intervention strategies

**DOI:** 10.1186/s12986-018-0306-7

**Published:** 2018-10-10

**Authors:** Jie-Hua Chen, Xu Lin, Cuihong Bu, Xuguang Zhang

**Affiliations:** 1 Science and Technology Centre, By-Health Co. Ltd, No. 3 Kehui 3rd Street, No. 99 Kexue Avenue Central, Science City, Luogang District, Guangzhou, 510000 China; 2 CAS Key Laboratory of Nutrition, Metabolism and Food Safety, Shanghai Institute of Nutrition and Health, Shanghai Institutes for Biological Sciences, University of Chinese Academy of Sciences, Chinese Academy of Sciences, Shanghai, 200031 China

**Keywords:** Advanced glycation end products (AGEs), Mobility, AGEs inhibitors, Dietary intervention strategies

## Abstract

Advanced glycation end products (AGEs), a group of compounds that are formed by non-enzymatic reactions between carbonyl groups of reducing sugars and free amino groups of proteins, lipids or nucleic acids, can be obtained exogenously from diet or formed endogenously within the body. AGEs accumulate intracellularly and extracellularly in all tissues and body fluids and can cross-link with other proteins and thus affect their normal functions. Furthermore, AGEs can interact with specific cell surface receptors and hence alter cell intracellular signaling, gene expression, the production of reactive oxygen species and the activation of several inflammatory pathways. High levels of AGEs in diet as well as in tissues and the circulation are pathogenic to a wide range of diseases. With respect to mobility, AGEs accumulate in bones, joints and skeletal muscles, playing important roles in the development of osteoporosis, osteoarthritis, and sarcopenia with aging. This report covered the related pathological mechanisms and the potential pharmaceutical and dietary intervention strategies in reducing systemic AGEs. More prospective studies are needed to determine whether elevated serum AGEs and/or skin autofluorescence predict a decline in measures of mobility. In addition, human intervention studies are required to investigate the beneficial effects of exogenous AGEs inhibitors on mobility outcomes.

## Background

### Overview of advanced glycation end products (AGEs)

#### What are they?

Advanced glycation end products (AGEs) are a heterogeneous group of compounds that are formed by non-enzymatic reactions between the carbonyl groups of reducing sugars and the free amino groups of proteins, lipids or nucleic acids. AGEs are produced in the Maillard reaction, which can cause browning, fluorescence and protein cross-linking, as well as the formation of flavour and aroma compounds [1]. AGEs can be formed within the body and can also originate from exogenous sources such as diet and smoking. Increased levels of AGEs are generated during the heat processing of food and the browning continues during storage. Various AGEs precursors are present and are formed during the initial, intermediate and final stages of the Maillard reaction and, depending on their composition and their molecular size, different AGEs compounds are created through a series of reactions involving enolisation, dehydration, cyclisation, fragmentation and oxidation. Such compounds include pyrraline, N^ɛ^-carboxymethyllysine (CML), N^ɛ^-carboxyethyllysine (CEL), pentosidine, argpyrimidine, derivatives of methylglyoxal (MG), hydroimidazolones derived from MG, glyoxal (GO), 3-deoxyglucosone (3-DG), arginine-derived N^δ^-ornithine and bis(lysyl)imidazolium derivatives, such as methylglyoxal-lysine dimer (MOLD) and glyoxal-lysine-dimer (GOLD) [1, 2]. Among these, CML, pentosidine, and furosine are considered as the common AGEs in foods and human plasma [3, 4], in which CML has been found as the most abundant AGEs in human plasma [5].

AGEs can be divided into three categories according to their ability to create cross-links on proteins and to show fluorescence: (1) fluorescent cross-linking AGEs such as pentosidine and crossline; (2) non-fluorescent cross-linking AGEs such as imidazolium dilysine cross-links, alkyl formyl glycosyl pyrrole (AFGP) cross-links and arginine-lysine imidazole (ALI) cross-links; (3) non-cross-linking AGEs such as pyrraline, CML and CEL [6]. Because of the complexity and heterogeneity of AGEs formation in vivo, the structures of cross-linked AGEs have not been completely determined.

Some researchers also classify AGEs as being toxic or non-toxic. Compounds such as CML, CEL and pyrraline are considered to be non-toxic AGEs. Toxic AGEs are usually derived from glyceraldehyde or glycolaldehyde. However, the structural identity of toxic AGEs remains unknown [7, 8]. In fact, recent studies have indicated the pathogenic role of some non-cross-linking AGEs (non-toxic) such as CML. For example, through an AGEs receptor, CML may affect cell signalling, may stress cells and may trigger cell injury, leading to pathological endothelial cell dysfunction and apoptosis of macrophages [9, 10].

#### Exogenous formation of AGEs

AGEs are naturally occurring chemicals in raw animal-origin foods, and cooking propagates and accelerates the generation of more AGEs within them. Studies have shown that dry heating results in the formation of more than ten to hundred times of new AGEs in foods as compared to the uncooked state [1]. For the food industry AGEs are greatly desirable owing to the profound effect of AGEs on safety and convenience as well as to enhance food flavour, colour and appearance, and thus increase food consumption [11, 12]. Dry heat, irradiation or ionization in modern food processing considerably promotes the formation of new AGEs [12–14]. Contents of AGEs in food are mainly calculated from measurements of a single marker (CML or MG derivatives), which is regarded as a major limitation considering levels of individual AGEs significantly vary in different foods [1, 4]. On the whole, AGEs contents in foods analysed for MG derivatives are associated with corresponding levels of CML. Animal-origin foods, as well as foods with a high level of fat and protein, contain relatively high contents of AGEs. On the other hand, low values of AGEs were found in uncooked and even cooked carbohydrate-rich foods such as fruits, vegetables, milk and whole grains [1]. The order of dietary AGEs levels in foods is found to be beef>cheeses>poultry>pork>fish>eggs [1].

AGEs enter the circulation together with other nutrients in food. Currently, there are limited data on absorption and bioavailability of AGEs [15]. Both animal studies and human studies confirmed that dietary AGEs are partially absorbed in the intestine (10–30%) [15–17]. The absorption rate differs between low molecular weight (LMW) AGEs and high molecular weight (HMW) AGEs. LMW AGEs may be relatively quickly absorbed, biotransformed, and excreted whereas HMW AGEs are absorbed more slowly and less efficiently due to insufficient degradation by gastrointestinal enzymes. About two thirds of the absorbed AGEs remain in the body for 3 days [17–20], resulting in increased oxidation stress, AGEs and potentially organ damage. The bioavailability of AGEs is largely influenced by factors such as diet, structures and gut environment etc. Global AGEs distribution in tissues were observed in animal studies, which have shown that more than half of the absorbed AGEs were bound in liver and kidney after 72 h, the rest could be found in heart, lung and spleen [15, 20].

Recent animal and human studies with an oral intake of an AGE-rich meal, labelled AGEs or specific AGEs have clearly demonstrated that dietary AGEs represent an important source for circulating AGEs and contribute to the in vivo AGEs pool under physiological conditions [17, 20–24].

Studies on dietary AGEs intakes in the general population are however scarce. The estimated average dietary AGEs intake in adults has been shown to be 15,000 AGEs kU/day [1, 25], which is considered to be high and inductive for inflammation. Diets rich in grilled or roasted meats, fats, and highly processed foods could achieve a level of AGEs higher than 20,000 kU/day [1]. The influence of dietary AGEs on the formation of endogenous AGEs is discussed in Section “Factors that Affect AGEs Formation in vivo” of this report.

Besides dietary AGEs, smoking is another source of exogenous AGEs. Scarce information, however, is found on sources of AGEs in smokers [26]. Cerami et al. (1997) reported that the water extracts of tobacco leaves contain reactive glycation products (glycotoxins) and formation of AGEs in vivo and in vitro was promoted by tobacco smoke, the process of which was concentration and time dependent [27]. The highly reactive glycotoxins can induce the formation of AGEs formation in hours whereas glucose or glucose-6- phosphate induced AGEs formation takes days to weeks [27]. Glycotoxins from cigarette enter the body via lung alveoli and then are transported to blood stream or lung cells where the formation of AGEs occurs by interacting with other glycation products [26, 28].

#### Endogenous formation of AGEs

The formation of AGEs endogenously is a part of the normal consequence of metabolism. However, this can be pathogenic if high levels of AGEs accumulate in tissues and the circulation. The formation of AGEs can be accelerated under certain conditions, such as hyperglycaemia, hyperlipidaemia and increased oxidative stress (OS). In fact, with aging and different diseases, elevated amounts of AGEs have been found in vivo [6, 29–32].

Generally, AGEs are formed physiologically in all tissues and body fluids, both intracellularly and extracellularly, when the carbonyl groups of reducing sugars react non-enzymatically with the free amino groups on proteins [33]. As this reaction occurs at a lower temperature and is less complex, compared with food production, there is less diversity of the AGEs compounds.

The research on AGEs in the human body has progressed dramatically during the last 20 years. One of the early examples is the identification of haemoglobin A1c (HbA1c), which is an Amadori rearrangement product, and results from the combination of glucose with the N-terminal valine residue of a haemoglobin B chain. It is measured clinically as an index of hyperglycaemia [34, 35].

**Fig. 1:**
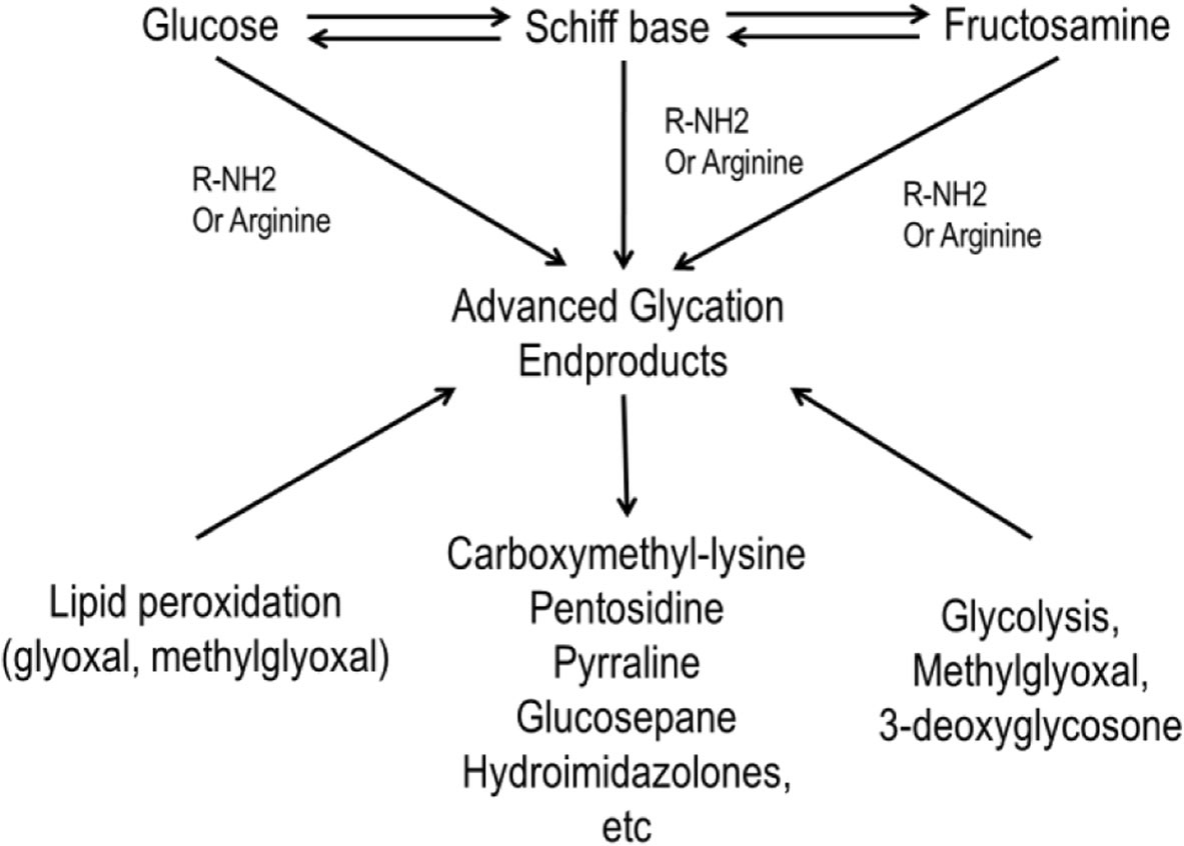
Formation of AGEs in vivo*. Adapted from Gugliucci and Menini (2014)* [104]

At least four types of process in the formation of AGEs under physiological conditions have been identified (Fig. 1) [15, 36].

1. Monosaccharide autoxidation (auto-oxidative glycosylation) or the degradation of saccharides unattached to a protein. This is an auto-oxidative pathway in which sugars give rise to reactive products by autoxidation.
2. Unstable Schiff base fragmentation, which is typically followed by the generation of a stable Amadori product.
3. Fructosamine (ketosamine) degradation.
4. The direct reaction of α,β-dicarbonyl compounds (oxoaldehydes) formed from the reducing carbohydrates and lipid peroxidation.

During formation of AGEs, the important sites of glycation are lysyl side chains, N-terminal amino groups and arginyl guanidine groups of proteins, guanyl bases of nucleotides and amino groups of phosphatidylethanolamine and phosphatidylserine [15]. Under physiological conditions, glucose is the most studied carbonyl precursor because it is the most commonly seen reducing sugar. The reaction between glucose and proteins in vivo is relatively slow. During the early stages of the Maillard reaction, Schiff bases are formed between reactive sugars and ɛ-amino groups. These can lead to an irreversible intramolecular rearrangement, which forms more stable Amadori products. The Amadori products undergo further structural changes through dehydration, oxidation and degradation to form highly stable AGEs, such as CML [37]. However, some highly reactive dicarbonyl compounds, such as GO, MG and 3-DG, are capable of rapid AGEs formation. They are generated by various pathways including glucose autoxidation, lipid peroxidation and interruption of glycolysis by reactive oxygen species (ROS). A large quantity of these dicarbonyl compounds induces so-called “carbonyl stress”, because they are highly reactive with both intracellular and extracellular proteins [7]. For example, 3-DG, which can be formed by the non-oxidative rearrangement and hydrolysis of Amadori products, reacts rapidly with protein amino groups to form AGEs such as imidazolone, pyrraline and CML [38, 39]. MG can be produced by the autoxidation of carbohydrates and lipid peroxidation in most glucose-metabolising cells, and reacts with the lysine residues on proteins to form CEL [40].

#### Factors that affect AGEs formation in vivo

As mentioned in Section “Exogenous Formation of AGEs”., the dietary AGEs load contributes to circulating AGEs and the AGEs pool in vivo, and hence dietary AGEs can affect endogenous AGEs formation through their effects on AGEs metabolism. Generally, AGEs are generated within the body in homeostasis. The rate of AGEs formation in vivo depends on a number of factors, including the nature and concentration of the substrate groups, the glycating agents, the half-life of the proteins, the availability of catalytic compounds, the OS or redox balance and the presence of inhibitors such as aminoguanidine and pyridoxamine.

The abilities of sugars to react with amino groups differ. The smaller sugar molecules with fewer carbon atoms are more reactive. The reactivity increases when there are more reactive open chains and furanose ring structures. For example, fructose is about 10-fold more reactive than glucose. The low reactivity of glucose, which is the predominant sugar in vivo, works as a native protection mechanism against the intracellular accumulation of AGEs and their precursors. As a result, the Maillard reaction is slow under normal metabolism [41]. However, under some sub-clinical conditions, such as hyperglycaemia and increased OS, elevated AGEs formation is triggered by increasing levels of reactive carbonyl intermediates, such as GO, MG and 3-DG. In addition, ROS are generated during the formation of AGEs, including stages such as the autoxidation of glucose, Schiff bases and Amadori adducts. When the level of ROS is elevated under unbalanced OS, a vicious cycle of AGE/ROS promotes more oxidation of lipids and glucose and accelerates the formation of AGEs in vivo [42].

The antioxidant systems in the body, including reducing agents, antioxidant enzymes and the detoxification system, can limit the level of AGEs precursors and reduce the generation of ROS. For example, the enzymes in the glyoxalase system, which carry out detoxification, can prevent the MG-mediated glycation and can convert most of the MG into harmless molecules such as lactate [43]. In the blood and body fluids, some early detoxification proteins, such as defensins, lactoferrin and lysozyme, are able to bind AGEs before their cellular uptake or cross-linking to other molecules [44, 45].

The most important mechanisms involved in the degradation of endogenous AGEs are extracellular proteolysis and the AGEs-receptor 1 (AGER1)-mediated intracellular uptake and degradation within cells [46]. For example, the degradation of AGEs by certain cells such as macrophages generates soluble AGEs, which can be excreted by the kidney [47].

**Fig. 2:**
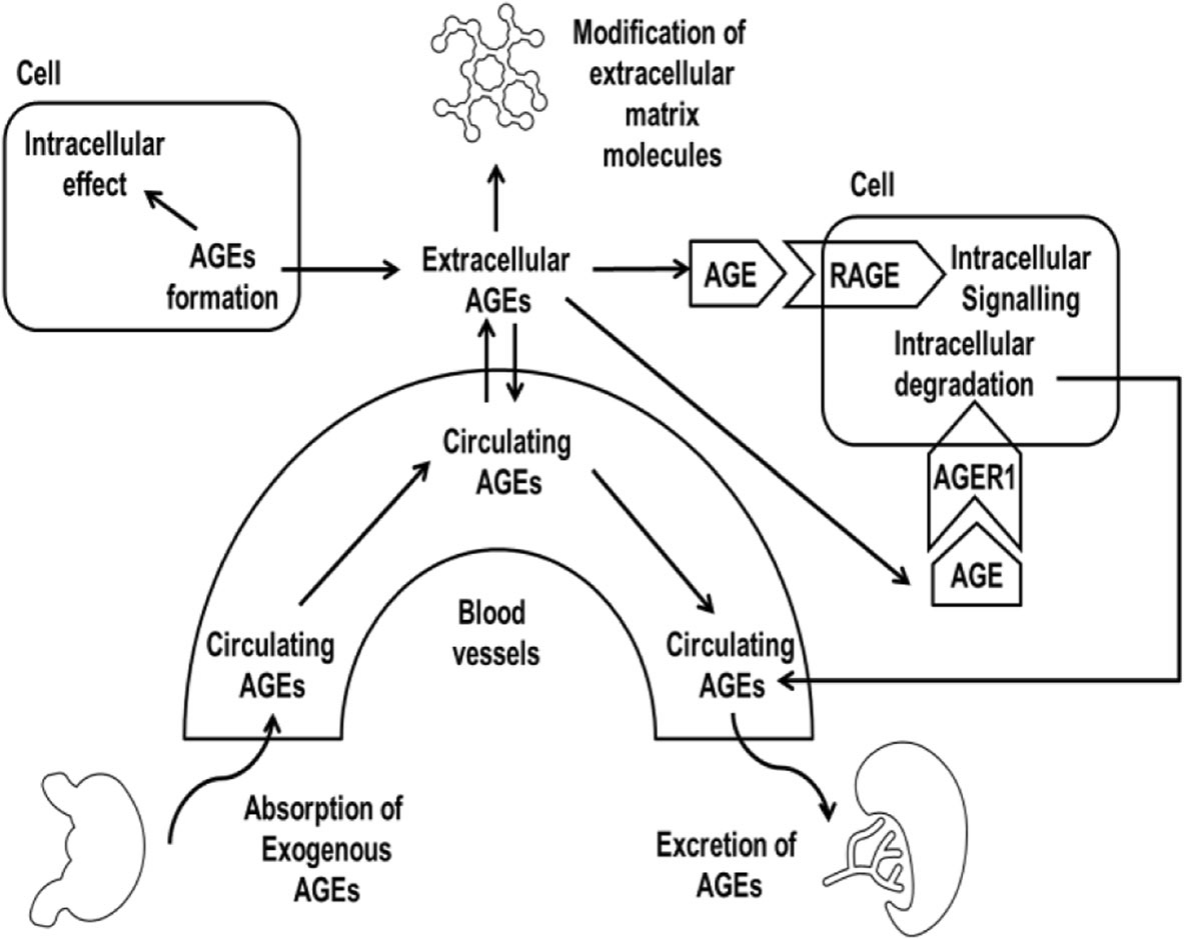
The cycle of endogenous and exogenous AGEs. *Adapted from Stirban* et al.*(2013)* [47]

In addition, as renal clearance works as an important factor for the level of circulating AGEs, it affects the accumulation of AGEs and the formation of endogenous AGEs. In fact, renal AGEs clearance can be affected by aging and the accumulation of AGEs in the kidney with renal impairment [48]. It can also be mediated by some functional compounds such as lysozyme, which may accelerate renal AGEs clearance [49]. In conclusion, AGEs are metabolised by innate defence and/or intracellular degradation after receptor-dependent uptake [45]. These protecting systems balance the endogenous formation of AGEs (Fig. 2) [40].

#### Direct impact of AGEs on plasma and extracellular proteins

The direct toxic effects of AGEs result from altering the structure and function of plasma and extracellular proteins by glycation and cross-linking. The glycation of proteins affects their normal function by disrupting their molecular conformation, interfering with their receptor function and altering their enzymatic activity. In addition, AGEs cross-link not only with proteins but also with other intracellular and extracellular molecules such as lipids and nucleic acids, which leads to structural and functional changes [6]. The alterations to the enzymatic proteolysis and degradation rate of some proteins such as elastin and collagen could lead to their accumulation in the extracellular matrix, which may result in changes in cellular adhesion and cell growth [50, 51]. This may decrease the elastic properties of both arterial and ventricular walls, causing the reductions in vascular and myocardial compliance that are characteristic of aging and diabetes [40].

Some long-lived structural proteins are more prone to AGEs accumulation because of their slow turnover. As these proteins provide the framework for most of the parenchymal organs, either in their fibrous form or in their basement membrane, their glycation and accumulation of AGEs could result directly in pathological outcomes, including renal function impairment, diabetic complications and affected bone health. For example, AGEs change the properties of collagen such as decreasing its solubility and changing its rigidity [52]. In the extracellular matrix region of the kidney, in which AGEs accumulate in collagen, these AGEs could cause changes in elasticity, ionic charge, thickness and turnover of basement membrane components, and hence could affect renal function [48]. Furthermore, the accumulation of AGEs in bone can affect bone strength and can lead to skeletal fragility by decreasing bone toughness and increasing stiffness [53, 54]. Non-enzymatic glycation of collagen may also exert a negative effect on bone remodelling and interfere with osteoblast differentiation [6, 55, 56].

### Impact of AGEs on inflammation, oxidative stress and insulin resistance

Apart from the direct impact of AGEs on proteins and the extracellular matrix, AGEs can also interact with specific cell surface receptors and hence alter cell intracellular signalling, gene expression, the production of ROS and the activation of several inflammatory pathways, including the release of pro-inflammatory cytokines, growth factors and adhesion molecules via activation of the nuclear factor kappa B (NF-κB) pathway (Table 1) [57].

**Table 1:** Receptors for AGEs

AGE Receptors	Cell Types	Function
RAGE	Monocytes/Macrophages, T-lymphocytes, endothelial cells, mesangial cells, fibroblasts, smooth muscle cells, neuronal cells	Endocytosis, signalling (cell activation), generation of ROS, inflammatory response
AGER1	Monocytes/Macrophages, T-lymphocytes, endothelial cells, mesangial cells, fibroblasts, smooth muscle cells, neuronal cells	Endocytic uptake and degradation of AGEs/AGE-modified proteins, protective against oxidative stress
AGER2	Monocytes/Macrophages, T-lymphocytes, endothelial cells, mesangial cells, fibroblasts, smooth muscle cells, neuronal cells	Signalling (cell activation), regulatory subunit of glucosidase II
AGER3	Monocytes/Macrophages, T-lymphocytes, endothelial cells, mesangial cells, fibroblasts, smooth muscle cells, neuronal cells	Signalling (cell activation)
SR-A	Monocytes/Macrophages, dendritic cells, endothelial cells	Endocytic uptake and degradation of AGEs/AGE-modified proteins and modified LDL
SR-B	Platelets, endothelial cells, epithelial cells, adipocytes, lymphocytes	Endocytic uptake and degradation of AGEs/AGE-modified proteins, cell adhesion, regulator of fatty acid transport
SR-BI	Tissues that are active in selective uptake of high density lipoprotein (HDL)	Selective uptake of HDL, endocytic uptake and degradation of AGEs
SR-E	Macrophages, endothelial cells, smooth muscle cells	Signalling, endocytic uptake and degradation of OxLDL
FEEL-1/FEEL-2	Monocytes/Macrophages, endothelial cells	Endocytic uptake and degradation of AGEs/AGE-modified proteins, hyaluronic acid and AcLDL

RAGE, receptor of advanced glycation end products; AGER1, AGER2 and AGER3, advanced glycation end product receptor-1, −2 and − 3; SR-A, scavenger receptor class A; SR-B, scavenger receptor class B; SR-BI, scavenger receptor class B Type I; SR-E, scavenger receptor class E; FEEL-1 and FEEL-2, link domain-containing scavenger receptor-1 and -2; OxLDL, oxidised LDL; AcLDL, acetylated LDL

The most well-studied AGEs receptor is the receptor of advanced glycation end products (RAGE), which is the main up-regulator of cell activation in response to the AGEs load. RAGE is a multi-ligand receptor, belongs to the immunoglobulin superfamily and has a highly charged, cytoplasmic domain. It recognises a range of ligands including AGEs, leukocyte integrin Mac-1, modified low density lipoprotein (LDL), DNA, RNA and S100 calcium-binding protein B (S100B) [58]. AGE–RAGE interaction triggers various intracellular signalling cascades, followed by the transcription of a range of genes involved in different biological systems, which perpetuates the inflammatory/pro-inflammatory signals [59]. Specifically, this axis stimulates Janus kinase/signal transducers and activators of transcription (JAK/STAT), p38 mitogen-activated protein kinase (p38 MAPK), extracellular signal-regulated protein kinases 1 and 2 (ERK 1/2) and c-Jun N-terminal kinase (JNK), which leads to the activation of transcription factors NF-κB and interferon-stimulated response elements (ISRE). This causes increased expression of cytokines, growth factors and adhesion molecules. Furthermore, AGE–RAGE interaction also stimulates the generation of ROS via the nicotinamide adenine dinucleotide phosphate (NADPH) oxidase pathway [47, 57, 59, 60].

In contrast, there are a number of AGEs receptors, such as the advanced glycation end product receptor (AGER) family and the scavenger receptor (SR) family, that mediate endocytosis, leading to the intracellular uptake and degradation of AGEs by their fusion with lysosomes [61–64]. Furthermore, AGEs peptides can be transferred to the renal system, whereas the receptors are recycled and available for further endocytosis processes [57, 65].

**Fig. 3:**
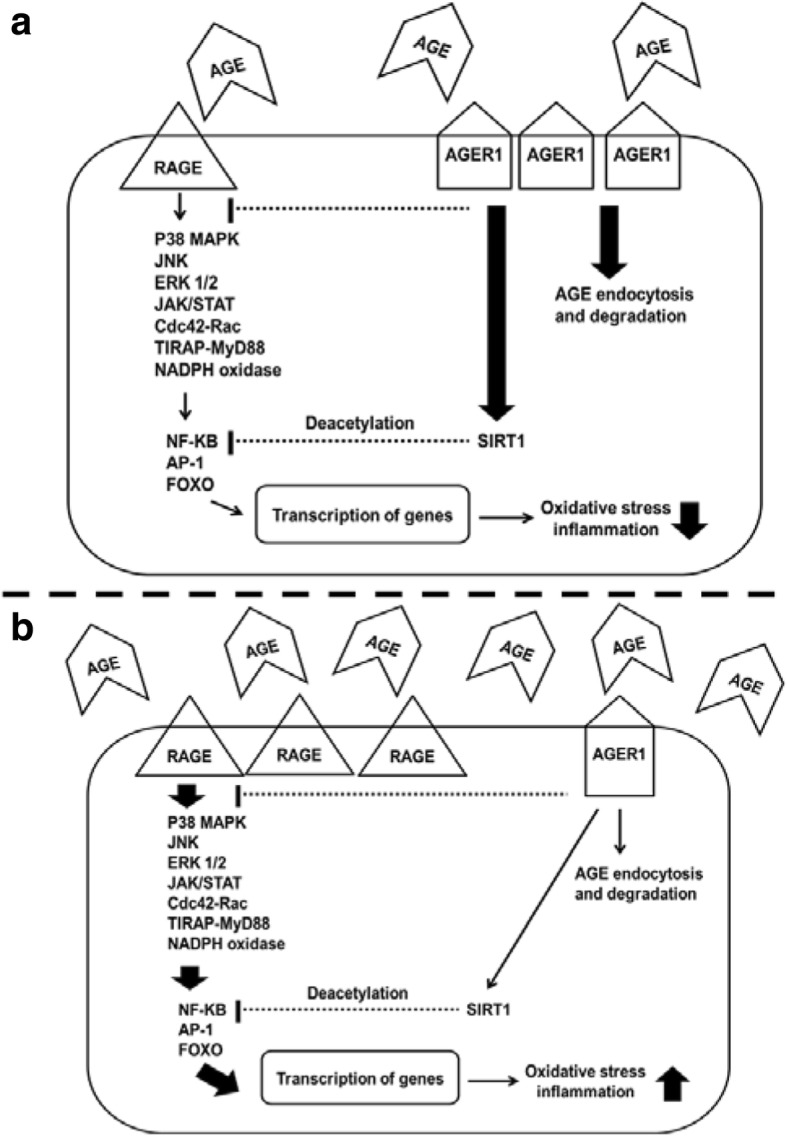
Scheme of the interaction of AGEs with RAGE and AGER1 under conditions with different AGE loads, **a**) a low AGEs burden, and **b**) an overload of AGEs. AGER1, AGE receptor 1; AP-1, activator protein 1; Cdc42-Rac, cell division control protein 42 homolog-Rac; ERK 1/2, extracellular signal-regulated protein kinases 1 and 2; FOXO, forkhead box protein O subclass; JAK/STAT, Janus kinase/signal transducers and activators of transcription; JNK, c-Jun N-terminal kinases; NADPH, nicotinamide adenine dinucleotide phosphate; NF-κB, nuclear factor kappa B; p38 MAPK, p38 mitogen-activated protein kinases; RAGE, receptor for AGEs; SIRT1, sirtuin-1; TIRAP-MyD88, toll-interleukin 1 receptor domain containing adaptor protein and myeloid differentiation primary response protein 88. *Adapted from Poulsen* et al.*(2013)* [15]

The expression level and the activation of AGEs receptors depend on the cell or tissue type (Table 1) and are regulated in response to the AGEs load, other metabolic changes, conditions such as hyperlipidaemia, aging and diabetes mellitus [66]. For example, in Fig. 3a, in response to conditions with a low AGEs burden, the expression of RAGE is down-regulated whereas the expression of AGER1 is up-regulated. As the RAGE signalling pathway leads to the activation of transcription factors NF-κB, activator protein 1 (AP-1) and forkhead box protein O subclass (FOXO), the down-regulated RAGE reduces the transcription of genes related to OS and inflammation. The up-regulation of AGER1 also inhibits these transcription factors through the sirtuin-1 (SIRT1) pathway. In addition, the increased expression of AGER1 may accelerate the intracellular degradation of AGEs, which results in an overall lower degree of OS and inflammation caused by AGEs. In Fig. 3b, when there is an AGEs burden, RAGE is up-regulated, leading to increased OS and inflammation. The prolonged high AGEs burden leads to down-regulation of AGER1, which, therefore, cannot exert strong inhibitory effects on RAGE signalling or reduce the levels of AGEs by their degradation [15]. Recent studies demonstrate that the interaction between AGEs and RAGE can cause cell migration and adhesion. For example, the activation of RAGE on monocytes can induce the migration of these cells into the sub-endothelial space [67]. Via interaction with integrin CD11b and their increased expression on endothelial cells, RAGEs can work as receptors for recruiting monocytes and neutrophils, and promoting the adhesion of leukocytes to the vessel wall [68, 69].

Oxidative Stress-induced cell signal transduction disturbances results in increased OS and disrupted antioxidant defense system, which is implicated in the development and persistence of Insulin Resistance (IR) [70]. Molecular mechanisms of IR involves an array of pathways including cell-specific redox regulation of protein kinases C (PKCs) and changes in the insulin signal pathways phosphatidylinositol 3-kinase (PI3K) and MAPK, suppressing protective survival systems AGER1 and SIRT1 [70–77]. High AGEs in muscle, insulin sensitive tissue, induce IR and activate NFκB by oxidative activation of PKC that phosphorylates regulatory serine residues on insulin receptor substrate-1 (IRS1) in the PI3K pathway [70, 71].

### Methods for measuring AGEs and cut-off values for different populations

Several analytical methods are available for measurement of circulating or tissue-bound AGEs, e.g., enzyme-linked immunosorbent assays (ELISA) [22], fluorescence spectroscopy [78], and mass spectrometry (MS)-based high-performance liquid chromatography (HPLC)/gas chromatography (GC) [79]. MS-based methods are often used in laboratories for the diagnosis and monitoring of age-related chronic diseases due to high sensitivity, reproducibility and accuracy. LC coupled with tandem mass spectrometric (LC–MS/MS) has been used to accurately quantify glycation adducts in plasma, urine, and dialysate samples in patients with uremia [80].

Tissue-bound AGEs are usually measured in the skin due to easy accessibility by skin autofluorescence (SAF), a simple and non-invasive technique [78], which has been validated against the gold standard method skin biopsies [81]. Validation studies indicated significant association of SAF with AGEs content in skin biopsies. Meta-analysis of three validation studies has shown that skin AGEs content attributed up to 76% of the variance in the SAF levels, suggesting that SAF can act as a biomarker of cumulative skin AGEs [78, 82–84]. Koetsier et al. (2010) conducted a cross-sectional study and provided reference values of SAF for healthy Caucasian control subjects over a broad age range [85].

SAF has been demonstrated to be potentially better predictor for the development of chronic complications and mortality in diabetes over time (5–10 years) than glycated haemoglobin A1c which reflects short-term glycemic status (3–6 months) [81, 82, 86, 87]. Smit et al. (2013) suggested that decision tree method could be used for early diabetes screening in risk groups [88]. According to previously published reference values, cut-off values for SAF would be ≥80th age percentile for age group < 50 years or ≥ 70th age percentile for age group ≥50 years [85, 88–90]. In addition, SAF values (> 2.0 AU over 5 years) has been revealed to be a significant marker for the induction and development of vascular complications that can predict CV risk and death [82, 87].

### Effects of AGEs on mobility

#### Effects of AGEs on chronic diseases

In healthy individuals, there are associations between serum AGEs levels and the risk factors for developing chronic diseases. Generally, the circulating AGEs levels are positively correlated with age, oxidative stress and insulin resistance [21, 25, 33]. Higher levels of AGEs have been found in healthy individuals with high dietary AGEs intakes than in individuals who eat foods containing fewer AGEs [91]. In recent studies, the serum concentrations of AGEs have been positively associated with a wide range of diseases, such as obesity, insulin resistance, diabetes, metabolic dysfunction, renal diseases, cardiovascular diseases (CVD), osteoporosis, rheumatoid arthritis, cognitive impairments and cancer [9, 31, 48, 66, 92–104]. There are direct pathological contributions of AGEs because of their protein cross-linking and accumulation. For instance, in kidney diseases, this could reduce the renal clearance of AGEs and could also increase endogenous AGEs formation [17, 105]. The increased levels of AGEs in vivo can be seen not only as an outcome of the development of these diseases and their relevant complications, but also as a cause of the pathogenesis of some diseases, such as diabetes [12]. This is generally related to the effects of AGEs on protein dysfunction, oxidative stress and inflammation.

Dietary AGEs have been shown to be correlated with serum levels of AGEs, inflammation markers, metabolic dysfunction and life expectancy [5, 21, 24, 25], suggesting that dietary AGEs are pathogenic, and predisposing the body to the development of CVD, diabetes and other chronic diseases, possibly via inducing systemic oxidative stress [21]. Animal studies demonstrated that AGEs supplementation promotes oxidative stress [21, 45, 106]. Similar results were observed in humans, implicating a correlation between dietary AGEs and oxidative stress, and thus increased risk of CVD, renal diseases and diabetes [25, 45]. Modern diet contains high levels of AGEs, resulting in excessive influx of AGEs into the circulation system, and thus enhancing the basal oxidant stress and inflammation [99, 107, 108]. As a result, β-cell functions are prone to be compromised, leading to insulin dysfunction and other diabetic complications. Studies on mice have demonstrated that reduction in dietary AGEs decreased oxidative stress and thus prevents or ameliorates type 1 and type 2 diabetes [109–111].

#### AGEs and mobility

Mobility underlies the ability to perform the basic activities of daily living that are necessary for independence and is a core indicator of health and function in aging [112]. In recent years, there has been increasing evidence that poor mobility outcomes are linked to in vivo AGEs levels [53, 113–117].

##### AGEs and bone health

Loss of bone mineral and/or bone mass is considered to be the major cause of age-related bone fractures. In fact, every year, more than 8.9 million fractures worldwide are caused by osteoporosis. As there is a lack of the initial symptoms of osteoporosis, many cases are diagnosed only after the first clinical fracture has occurred [118].

Osteoporosis is a progressive skeletal disease, the aetiology of which is attributed to various factors such as endocrine, metabolic and mechanical factors. It is characterised by a systemic impairment of bone mass, density and strength and structural deterioration of the bone microarchitecture, which leads to enhanced bone fragility and an increased risk of fractures. Osteoporosis can occur at any age, but is predominantly found in certain populations such as the elderly and with diseases such as diabetes [119].

Bone is a permanently remodelling organ; it is continually renewed in a complex process of formation by osteoblasts and resorption by osteoclasts. AGEs accumulated in the bone matrix may result in suppressed bone formation as evidenced by an animal study, which showed that significant reductions in mineral apposition rate, mineralized surface per bone surface and bone formation rate were observed in rats with an autograft implant containing AGEs [120]. Bone matrix homeostasis is heavily influenced by nutritional and hormonal factors. Recent studies have proposed non-enzymatic protein glycation as a new factor that affects bone remodelling. The glycation pathway has been implicated as a strong contributor to age-related diseases such as osteoporosis [121]. In support of this concept, increased serum levels of AGEs, such as pentosidine and CML, are found in individuals with osteoporosis [114]. Recent findings also provide important evidence that bone proteins are affected by AGEs modification. Furthermore, these AGEs can influence both osteoclasts and osteoblasts [55, 56, 115].

Type I collagen, contributing to 85% of the bone matrix, is the most abundant protein of bone and has an exceptionally long lifetime, making them susceptible to modification by AGEs [122]. Adverse changes in the collagen network of the bone matrix occur as people age and such changes may lead to deterioration in bone quality. For example, a significantly higher level of pentosidine has been found in the collagen in both cortical and trabecular bone for individuals aged over 65 years [121]. When collagen is cross-linked with AGEs, it has decreased solubility and becomes highly resistant to proteolytic degradation, which consequently leads to stiff collagen with disrupted function. The increased level of AGEs cross-links between collagen molecules is one of the dominant factors affecting the integrity of the collagen network in bone. Furthermore, it has been demonstrated that AGE-modified collagen boosts the intracellular release of ROS, interferes with the adhesion of osteoblastic cells to the matrix and inhibits osteoblastic differentiation and proliferation [123–125].

It is known that AGEs affect osteoblast differentiation and proliferation by binding to their receptors [126]. Similarly (Fig. 3), in these cells, the binding of AGEs to RAGE activates NF-κB, resulting in increased expression of cytokines, growth factors and cell adhesion molecules. This initiates inflammatory processes and elicits oxidative stress, leading to abnormal osteoblast function and bone remodelling disorder [115]. For example, there is reduced synthesis of Type I collagen and osteocalcin by human osteoblast-like cells after they have been treated with AGE-modified bovine serum albumin (BSA) [127]. In human osteoblast primary cell culture, there is a dose-dependent effect of the AGE pentosidine on osteoblast function [56]. In primary osteoblasts derived from fetal rat, AGE-collagen suppressed mature bone nodule formation, one of the osteoblastic parameters [122]. These studies have shown that AGEs impaired both bone matrix production and mineralization of osteoblasts.

Although contribution of AGEs to osteoblasts differentiation and function has been well documented in vitro [125, 128–131], their roles in osteoclast activity and differentiation remain mostly elusive. Different AGEs receptors including RAGE were found expressed in both osteoclast progenitors and mature osteoclasts. AGEs might interact with specific cell-surface receptors to interfere with the process of osteoclastic differentiation and activity [131]. In vitro resorption assay on AGE-modified mineralized matrices revealed that AGEs impaired the structural integrity of bone matrix proteins and the osteoclastic differentiation process, resulting in decreased osteoclast-induced bone resorption. Osteoclastogenesis was inhibited in vitro in the presence of AGEs, most likely by impairing the process of osteoclast progenitors into pre-osteoclastic cells, possibly mainly by a RAGE-dependent manner.

AGE-induced chronic inflammation in bone can be seen as a pathogenetic factor in osteoporosis [116]. It can significantly affect bone turnover, influencing the intrinsic balance of bone mineralisation and resorption [132]. By stimulating the expression of pro-inflammatory cytokines, such as interleukin (IL)-1, IL-6, tumour necrosis factor (TNF)-α and leukaemia inhibitory factor (LIF), AGEs may work as pro-osteoporotic mediators, regulating both osteoblasts and osteoclasts [133, 134]. For example, IL-1, IL-6 and TNF-α work as stimulators for bone resorption by promoting osteoclast activity and inhibiting bone formation [116]. Some modulator-like LIF has a dose–response role, with a high concentration inducing bone resorption and a lower dose promoting bone formation [135, 136]. However, although these studies have demonstrated the pathophysiological effects of these cytokines on bone cells, their exact roles (mechanistic pathways) in the development of osteoporosis have not been elucidated. Furthermore, a growing number of clinical studies have demonstrated not only the concomitance of regional osteoporosis with regional inflammation, but also the association between systemic osteoporosis and events of systemic inflammation, which exposes osteoporosis to the effects of general in vivo AGEs [116].

**Fig. 4:**
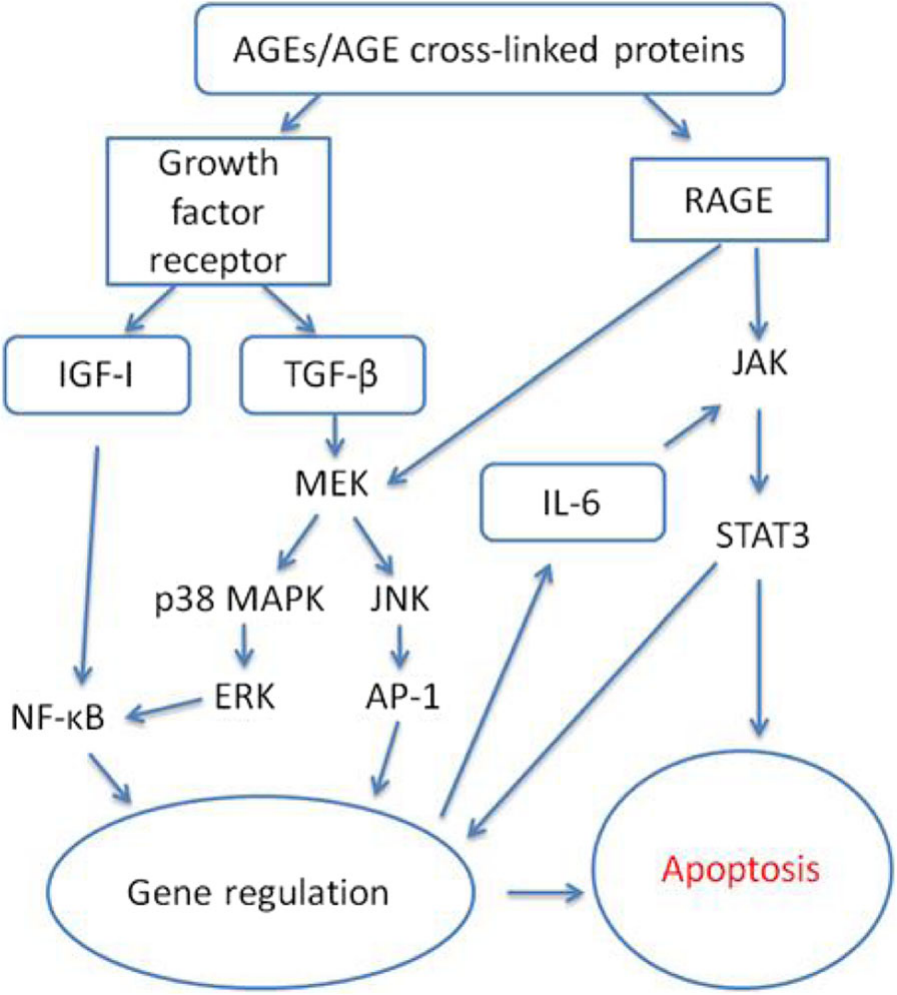
Effect of AGEs on apoptosis signalling. AP-1, activator protein 1; ERK, extracellular signal-regulated protein kinases; IGF-I, insulin-like growth factor I; IL-6, interleukin-6; JAK, Janus kinase; JNK, c-Jun N-terminal kinases; MEK, mitogen-activated protein kinase; NF-κB, nuclear factor kappa B; p38 MAPK, p38 mitogen-activated protein kinase; RAGE, receptor for AGEs; STAT3, signal transducers and activators of transcription 3; TGF-β, transforming growth factor-β

The detrimental effects of AGEs on osteoblast function also include increased apoptosis signalling by the activation of RAGE and other receptors such as growth factor receptors [55]. Figure 4 shows that this involves the regulation of various autocrines and paracrines, such as insulin-like growth factor I (IGF-I), IL-6 and transforming growth factor-β (TGF-β) [119, 123, 130, 137, 138]. For example, it has been reported that the physiological level of CML-cross-linked collagen can stimulate apoptosis in various osteoblastic cell cultures and that the signalling is mediated through RAGE by stimulating both p38 MAPK and JNK [114, 126]). The enhanced osteoblast apoptosis by AGEs contributes to the mechanisms of the development of osteoporosis [139].

##### AGEs and joint health

Osteoarthritis (OA) is one of the most frequent chronic diseases of the elderly. It is characterised by the softening, ulceration and disintegration of articular cartilage, as well as by the formation of outgrowths of bone and cartilage at the joint margins [96]. It is a major source of pain, disability and socioeconomic cost worldwide [140]. The exact pathophysiological mechanisms of OA are not completely clear; they can be complex and multifactorial, including genetic, biological and biomechanical components. However, age has been recognised as one of the main risk factors for the development of OA [141]. In fact, the majority of people older than 60 years have OA in at least one joint [142] and the development of OA is strongly correlated with chronological age [143]. Therefore, age-related changes in articular cartilage are likely to play a role in the aetiology of OA.

One of the major age-related changes in articular cartilage is increased levels of cross-linked AGEs. From age 20 onward, AGEs accumulate in collagen and proteoglycans in articular cartilage. The prevalence of OA increases with age and coincides with the accumulation of AGEs in articular cartilage [144, 145]. Recent studies have found that the increased AGEs levels can negatively modify articular cartilage by increasing its stiffness, elevating chondrocyte-mediated proteoglycan degradation, decreasing proteoglycan synthesis and inducing the degradation of the extracellular matrix (ECM) of cartilage [141, 146, 147]).

The ECM of cartilage is degraded by matrix metalloproteinases (MMPs), which can be induced by cytokines. The accumulation of AGEs in articular cartilage may increase oxidative stress and stimulate the expression of cytokines, which can affect the turnover of the ECM [148]. A study in which mice were fed a high AGEs diet showed the up-regulation of syndecan-4 and MMP-3; this was proposed for the activation of aggrecanase II (a disintegrin and metalloproteinase with thrombospondin motifs 5, ADAMTS-5), which is a critical phenomenon in the development of OA [149, 150]. In human C28/I2 chondrocytes, the blocking of RAGE prevented the increase in ADAMTS-5, syndecan-4, MMP-1 and MMP-3 in vitro after treatment with AGE-modified BSA [150]. As articular cartilage is one of the tissues containing the greatest amounts of AGEs in the body, AGE-related negative effects on the synthesis and degradation of proteoglycan in articular cartilage and ECM turnover are likely to be important contributors to the development of OA [141, 151, 152]. However, the causative link between AGEs and OA, the pathophysiological mechanisms and the pathways by which dietary AGEs affect joint tissue such as cartilage are not yet completely clear.

##### AGEs and skeletal muscle

Sarcopenia, as loss of muscle mass, strength and endurance, is an important factor causing mobility difficulties such as low strength, decreased lower extremity performance, slow walking speed and physical disability [113, 153]. It is estimated that there could be an average of 5% loss of muscle mass every 10 years after 40 years of age, with a decrease in muscle cross-sectional area, loss of muscle fibre and fibre atrophy, and that this may get worse after age 65 [154, 155]. In fact, sarcopenia occurs to some degree as a consequence of aging in all individuals, but can be accelerated by a variety of factors including inactivity, poor nutrition, increased oxidative stress and chronic disease conditions [155].

Recently, it has been hypothesised that AGEs could play a role in the pathogenesis of sarcopenia through cross-linking tissues in skeletal muscle, AGE–RAGE-mediated inflammation and endothelial dysfunction in the microcirculation of skeletal muscle [156, 157]. Both animal studies and human studies have reported increased levels of AGEs in skeletal muscle with aging [117, 157–159]. The accumulated AGEs may contribute to increased stiffness in muscle tissue, similar to that in articular cartilage, and may also reduce the viscoelastic properties of muscle and hence impair muscle function. The AGEs cross-linked with connective tissue surrounding muscle fibres may contribute to the decline in muscle function with aging [157]. Furthermore, when AGEs accumulate in endothelial cells, they up-regulate inflammation through RAGE and contribute to endothelial dysfunction, which leads to loss of muscle mass and strength [156]. In fact, some studies have reported that the elevated serum AGEs levels are associated with sarcopenia-related outcomes, such as poor grip strength, slow walking speed and increased muscle weakness [153, 160].

To summarise, AGEs accumulate in bones, joints and skeletal muscles. They may play important roles in the development of osteoporosis, osteoarthritis and sarcopenia with aging. More prospective studies are needed to determine whether elevated serum AGEs levels predict a decline in mobility measures. In addition, intervention studies are required to investigate the beneficial effects of low endogenous AGEs levels and the effects of the accumulation of AGEs on mobility outcomes.

### Current therapeutic and nutritional anti-AGEs approaches

#### Therapeutic approaches

As AGEs contribute to the development of chronic diseases such as diabetes, cardiovascular disease and renal impairment by their impacts on oxidative stress and inflammation, they are considered to be promising drug targets for a potential therapeutic approach, which aims to prevent diabetic or other pathogenic complications. In fact, it has been reported that such pharmaceutical interventions effectively prevent and treat diabetic and other pathogenic complications [161–163]. A large number of compounds have been reported to play a potential role against AGEs [164], e.g. aminoguanidine [163, 165, 166], vitamins (e.g. thiamine, pyridoxamine, citric acid) [167–169], anti-inflammatory drugs with anti-glycation properties (e.g. aspirin, tenilsetam) [170, 171], antidiabetic drug (thiazolidinediones) [172], anti-hypertensive drugs with AGEs inhibition activity (e.g. angiotensin converting enzyme inhibitors) [173], HMA-CoA reductase inhibitors (Statins) [174], antioxidant agents (carnosine, flavonoids, curcumin) [175, 176], and chelators with AGE inhibition properties (carnosine, pyridoxamine) [177]. Results obtained from preclinical evaluation studies demonstrated anti-AGEs activity of these compounds but a clear mechanism of action has not been elucidated [79, 164]. The effects of many of these substances require further validation by clinical studies. We will discuss the most relevant compounds in this section.

Both synthetic and natural compounds have been evaluated as inhibitors against the formation of AGEs and their cross-linking to proteins. There are different inhibitory mechanisms, including inhibiting AGEs formation, accelerating the catabolism of existing AGEs or AGEs cross-links and blocking the biological response of AGEs [167]. Aminoguanidine, a synthetic compound, exhibited AGE inhibitory effect in rats [163]. The possible mechanism is that a nucleophile hydrazine group in aminoguanidine binds to carbonyl groups, leading to the decrease of accumulation of AGE-mediated collagen cross-linking [163]. Following a series of preclinical and small clinical studies [161, 178–188], two large clinical trials the ACTION trial and the ACTION-II trial [165, 166] were conducted to target at the anti-AGEs effects of aminoguanidine in patients with diabetes. An expected significant beneficial effect of aminoguanidine in preventing the progression of disease, however, failed to be observed. In ACTION-II study, patients treated with aminoguanidine frequently reported side effects, which included flu-like symptoms, liver abnormalities, gastrointestinal disorders and anemia [166]. Considering the toxic and potential side effects of synthetic molecules, natural products are preferable.

With respect to the in vivo mechanisms of AGEs formation, antioxidants can protect against the glycation of proteins that is caused by the exposure of protein to glucose under oxidative conditions. However, studies confirm the inhibitory function of antioxidants only on the formation of glycoxidation products but not for the glycation of proteins [176]. Conflicting results have been obtained when antioxidants, such as α-tocopherol, retinol and ascorbic acid, have been used for treating diabetic complications such as cataracts [189–191]. Only flavonoids have shown promising evidence that demonstrates their inhibitory effect on AGEs formation and the prevention of some complications of diabetes [192–195].

Some metal chelators may also indirectly inhibit AGEs formation by blocking ROS and free transition metal ions, which have been recognised as key players in advanced glycation [167]. They are applied mainly as drugs that are commonly used for the treatment of diabetic complications. Some natural compounds, e.g. citric acid, can be considered to be an AGE inhibitor because of its non-specific metal-chelating activity. In addition, pyridoxamine (a natural derivative of vitamin B6), as a dietary supplement, proved to be a potent inhibitor of AGEs formation with a stronger effect than aminoguandine [164]. Pyridoxamine can chelate metal ions that catalyse Amadori reactions, reduce the generation of dicarbonyl intermediates, and hence can inhibit the formation of AGEs [196]. It was revealed in preclinical studies as an effective agent in prevention of kidney and cardiovascular disease, reducing cross-linking of collagen, decreasing CML and CEL levels, and regulating oxidative stress [197–199]. Clinical studies on pyridoxamine, however, failed to demonstrate the anti-AGEs effects [200, 201].

Another vitamin derivative, bentotiamine, is a fat-soluble derivative of thiamine, found to be able to prevent diabetes-induced CML in rats [164, 202]. The plausible mechanisms appear to be the activation of transketolase, which targets the precursors of AGEs towards the activation of pentose-5-phosphate pathway, blocking other pathways involved in production of AGEs [203, 204]. However, conflicting evidences have been obtained from human studies about anti-AGEs effect of benfotiamine. An RCT study on patients with T2DM and nephropathy treated with benfotiamine found no significant impact in levels of CML, CEL, 5-hydro-5-methylimidazolone and other inflammatory markers [205]. Similar results were also observed in another RCT, in which inflammatory markers and AGEs levels were found no difference between treated patients and controls [206]. In contrast, a study revealed that benfotiamine was able to significantly reduce serum markers of endothelial dysfunction and AGEs levels [207].

Some AGEs inhibitors directly scavenge the reactive carbonyls. These molecules have one or more nucleophilic centres that display the capacity to trap different carbonyls [167]. In addition to pharmaceutical agents such as aminoguaidine, natural compounds such as thiamine (vitamin B1), benfotiamine, pyridoxamine and some natural polyphenols, have demonstrated effective inhibitory effects on the glycation of proteins by scavenging carbonyl species both in vitro and in vivo [203, 208–210]. For example, theaflavins from black tea and epicatechins from green tea effectively trapped MG in vitro, and have been suggested as potential AGEs inhibitors for in vivo studies [211].

Besides exogenous AGEs inhibitors, other inhibitors that are involved in the catabolism of AGEs mainly serve as physiological reducing agents, antioxidant enzymes and agents in the detoxification system. As discussed in Section “Endogenous Formation of AGEs”., they work as a defense system, balancing the AGEs in vivo pool.

Furthermore, AGEs breakers, which aim to break the AGEs cross-links, have been proposed. However, although the mechanism is very promising, as the recovery of oxidised proteins would be expected, the real effects of the proposed AGEs breakers are unlikely to be the result of the cleavage or the reversal of existing protein–AGE cross-linking; instead, they will have more direct effects on the formation of AGEs, such as their antioxidant and chelating effects and their reaction mechanism with dicarbonyl intermediates in the Maillard reaction [167].

In addition, there are AGEs inhibitor pharmaceuticals that target the RAGE–AGE axis. They work as either antagonists of RAGE or antagonists of circulating AGEs [212–215]. Interestingly, some antibacterial proteins, in particular lactoferrin and lysozyme, bind to AGEs with high affinity, before cellular uptake or their cross-linking to proteins [44, 45, 216]. Lysozyme also accelerates the renal clearance of AGEs and suppresses intracellular AGE-mediated signalling [49]. As lactoferrin is a milk protein and lysozyme has already been applied in infant formula, these two proteins may be considered to be functional ingredients that can be added to food as AGEs inhibitors [217, 218]. In fact, a registered patent (US 5891341 A; Li et al., 1999) uses the similar molecular domains of lactoferrin and lysozyme to remove AGEs.

#### Dietary approaches

Cooking strategies such as brief heating time, low temperatures, high moisture, and/or exposure to an acidic solution are effective in suppressing generation of new AGEs in food [1, 4]. Cooking methods involving high temperature such as frying, grilling, roasting and broiling propagate the production of dietary AGEs compared to low temperature cooking methods such as boiling, steaming, poaching and stewing. For example, AGEs detected in roasted or broiled chicken increase about four times than the same piece meat cooked by poaching or steaming [1, 4]. Preexposure to acidified environment (marinades such as lemon juice and vinegar) can be encouraged to inhibit the new formation of dietary AGEs. For example, unmarinated beef contains more than half the amount of AGEs than marinated beef [1].

The dietary AGEs database indicated that cuisines involving high consumption of fish, legumes, whole grains, low-fat milk products, fruits and vegetables, such as Mediterranean and Asian cuisines, are more favourable in reducing dietary AGEs as compared to diets featured with solid fats, fatty meats, full-fat dairy products and highly processed foods. These recommendations are in good agreement with dietary guidelines set by organizations such as the American Heart Association [219], the American Institute for Cancer Research [220], and the American Diabetes Association [221].

Dietary AGEs interventions can minimise the absorption of AGEs via the gastrointestinal tract and reduce the levels of circulating AGEs. Therefore, long term intervention with a low AGEs diet may also reduce the pool of AGEs in vivo. Table 2 lists human intervention studies in which a low dietary AGEs intake was compared with a high AGEs load. The dietary AGEs levels correlated with serum concentrations of AGEs in both healthy individuals and people with different disease conditions. Reduced serum AGEs levels were associated with improvements in levels of inflammatory markers and mediators, such as vascular cell adhesion molecule 1 (VCAM-1), NF-κB and TNF-α. These low AGEs diets may provide an important adjunct to interventions directly towards the inhibition of endogenous AGEs [40].

**Table 2:** Human intervention studies with low dietary AGE intakes

Population	Intervention	Affected AGEs	Affected AGE Receptors and Other Markers	AGE Measure Method	Country (Year)	Reference
Healthy	CML 2.2 mg/day vs 5.4 mg/day	↓Serum CML	↑ Vitamin C	GC–MS/MS	France (2010)	[99]
Healthy	CML 26 mg/meal vs 75.4 mg/meal	↓Serum CML		ELISA	Germany (2006)	[108]
Healthy	CML < 5500 kU/day vs > 13,000 kU/day	↓Serum CML, MG-derivatives	↓ VCAM-1, 8-isoprostanes, PBMCs, TNF-α, mRNA AGER1 and mRNA RAGE	ELISA	USA (2009)	[91]
Obese	CML 3302 kU/day vs 14,090 kU/day	↓Serum CML	↓ Urine 8-isoprostanes	ELISA	Australia (2011)	[225]
Diabetic	AGE intake decreased by 50% vs usual diet	↓Serum CML, MG-derivatives	↓ PBMCs TNF-α, NF-κB acetylation, and mRNA RAGE; ↑ mRNA AGER1, mRNA SIRT1 and circulating adiponectin	ELISA	USA (2011)	[46]
Diabetic	CML 3670 kU/day vs 16,300 kU/day	↓Serum CML	↓ AGE-modified LDL	ELISA	USA (2004)	[226]
Diabetic	7 U CML/mg protein vs 1617 U CML/mg protein	↓Serum CML		ELISA	USA (1997)	[17]
Diabetic	CML 2750 kU/meal vs 15,100 kU/meal	↓Serum CML	↓VCAM-1	ELISA	Germany (2007–2008)	[227–230]
Renal failure	CML 5500 kU/day vs 17,000 kU/day	↓Serum CML	↓ AGE-modified LDL, VCAM-1	ELISA	USA (2003–2004)	[23, 101]
Chronic kidney disease	CML < 5500 kU/day vs > 13,000 kU/day	↓Serum CML	↓ VCAM-1, 8-isoprostanes, TNF-α	ELISA	USA (2009)	[91]

AGER1, AGE receptor 1; CML, Nɛ-carboxymethyllysine; ELISA, enzyme-linked immunosorbent assay; GC–MS/MS, gas chromatography–tandem mass spectrometry; MG, methylglyoxal; NF-κB, nuclear factor kappa B; PBMCs, peripheral blood mononuclear cells; RAGE, receptor of advanced glycation end products; SIRT1, sirtuin-1; TNF-α, tumour necrosis factor alpha; VCAM-1, vascular cell adhesion molecule 1

## Conclusion

### Further considerations

As discussed above, intervention studies with low dietary AGEs intakes have been conducted in healthy individuals and patients with different diseases, measuring serum AGEs levels, inflammatory status and other AGEs markers in PBMCs, such as mRNA RAGE, mRNA AGER1, TNF-α and NF-κB. Furthermore, the effects of AGEs inhibitors, as pharmaceutical applications, have also been investigated in human studies that targeted diabetic complications. However, only a limited number of studies have focused on measuring the effects of low AGEs levels or AGEs inhibitors on mobility, although many observational human studies and in vitro studies have reported the correlation of AGEs with and the contribution of AGEs to mobility, particular in diseases such as osteoporosis, cartilage degradation, osteoarthritis and sarcopenia [56, 96, 113, 114, 124, 125, 130, 146, 147, 150, 153, 157, 222].

It should be noted that there is insufficient information from previous animal and human studies for use as a reference to determine the intervention period. Although serum AGEs levels can be easily affected by a lower AGEs diet or AGEs inhibitors, it may take longer to see the changes in certain organs or tissues, as a result of a reduction in AGEs accumulation. For example, the intervention period for the use of anti-AGE drugs in rats varied from 10 to 32 weeks, when AGEs levels in the kidney were measured [107, 223, 224]. Therefore, a long intervention period in which to observe changed AGEs levels in skin, bones, joints and muscles can be expected.

More prospective studies are needed to determine whether changed serum AGEs and/or skin autofluorescence predict different mobility measures. In addition, human intervention studies are required to investigate the beneficial effects of exogenous AGEs inhibitors on mobility outcomes.

## Abbreviations

3-DG: 3-deoxyglucosone
AcLDL: Acetylated LDL
AFGP: Alkyl formyl glycosyl pyrrole
AGER1: Advanced glycation end product receptor-1
AGER2: Advanced glycation end product receptor-2
AGER3: Advanced glycation end product receptor-3
AGEs: Advanced glycation end products
ALI: Arginine-lysine imidazole
AP-1: Activator protein 1
Cdc42-Rac: Cell division control protein 42 homolog-Rac
CEL: N^ɛ^-Carboxyethyllysine
CML: N^ɛ^-carboxymethyllysine
CVD: Cardiovascular diseases
ELISA: Enzyme-linked immunosorbent assays
ERK 1/2: Extracellular signal-regulated protein kinases 1 and 2
FEEL-1 and FEEL-2: Link domain-containing scavenger receptor-1 and -2
FOXO: Forkhead box protein O subclass
GC: Gas chromatography
GO: Glyoxal
GOLD: Glyoxal-lysine-dimer
HMW: High molecular weight
HPLC: High-performance liquid chromatography
IGF-I: Insulin-like growth factor I
IL-6: Interleukin-6
IR: Insulin resistance
ISRE: Interferon-stimulated response elements
JAK/STAT: Janus kinase/signal transducers and activators of transcription
JNK: c-Jun N-terminal kinase
LDL: Low density lipoprotein;LIF: leukaemia inhibitory factor
LMW: Low molecular weight
MG: Methylglyoxal
MMPs: Metalloproteinases
MOLD: Methylglyoxal-lysine dimer
MS: Mass spectrometry
NADPH: Nicotinamide adenine dinucleotide phosphate
NADPH: Nicotinamide adenine dinucleotide phosphate
NF-κB: Nuclear factor kappa B
OA: Osteoarthritis
OS: Oxidative stress
OxLDL: Oxidised LDL
p38 MAPK: p38 mitogen-activated protein kinase
PBMCs: Peripheral blood mononuclear cells
PKC: Protein kinase C
RAGE: Receptor of advanced glycation end products
ROS: Reactive oxygen species
SAF: Skin autofluorecence
SIRT1: Sirtuin-1
SR-A: Scavenger receptor class A
SR-B: Scavenger receptor class B
SR-BI: Scavenger receptor class B Type I
SR-E: Scavenger receptor class E
STAT3: Signal transducers and activators of transcription 3
TGF-β: Transforming growth factor-β
TIRAP-MyD88: Toll-interleukin 1 receptor domain containing adaptor protein and myeloid differentiation primary response protein 88
TNF-α: Tumour necrosis factor alpha
VCAM-1: Vascular cell adhesion molecule 1
